# Influence of running speed, inclination, and fatigue on calcaneus angle in female runners

**DOI:** 10.3389/fphys.2025.1505263

**Published:** 2025-04-09

**Authors:** Nina Verdel, Neža Nograšek, Miha Drobnič, Irinej Papuga, Vojko Strojnik, Matej Supej

**Affiliations:** ^1^ Faculty of Sport, University of Ljubljana, Ljubljana, Slovenia; ^2^ Department of Communication Systems, Jozef Stefan Institue, Ljubljana, Slovenia; ^3^ Deparment of Vascular Diseases, University Medical Centre Ljubljana, Ljubljana, Slovenia

**Keywords:** biomechanics, eversion, inversion, injury prevention, running related injury, 3D kinematics

## Abstract

Running is a popular form of physical activity with significant health benefits, but improper technique can lead to running-related injuries. This study investigates the influence of running speed, incline, and fatigue on calcaneus eversion/inversion angle at heel strike, maximum eversion angle, and range of motion, factors associated with lower limb injuries. Fifteen injury-free female runners participated in this study. Kinematic data were collected using a 3D motion capture system with reflective markers placed directly on the skin through specially modified running shoes. The runners performed treadmill trials at varying speeds (10, 12, and 14 km/h) and inclines (0°, 5°, and 10°), both before and after a fatigue-inducing 30-min run. The results indicate that higher speeds were associated with an increase in inversion angle at heel strike (*p* = 0.05) and range of motion (*p* = 0.02 before fatigue), both of which are linked to chronic ankle instability and Achilles tendinopathy. Running at an incline reduced both maximum eversion angle (*p* = 0.002 after fatigue) and range of motion (p = 0.003 after fatigue), suggesting a protective effect against excessive eversion. Fatigue increased range of motion (*p* = 0.05), which is a risk factor for instability and overuse injuries. These findings suggest that running at higher speeds and in a fatigued state may increase the likelihood of injuries due to increased range of motion, whereas incline running may mitigate this risk by reducing excessive eversion and range of motion. Understanding these biomechanical changes can inform injury prevention strategies for runners.

## 1 Introduction

Physical activity is essential for promoting health, enhancing wellbeing, and increasing life expectancy by reducing the risk of chronic diseases such as cardiovascular disease, type 2 diabetes, and cancer (Durstine et al., 2013). Among various forms of exercise, running is a highly accessible and cost-effective option that most individuals can engage in without special preparation. Research shows (Pedisic et al., 2020) that running significantly lowers the risk of all-cause mortality (27%), cardiovascular disease (30%), and cancer (23%) when compared to a sedentary lifestyle. However, while running offers considerable health benefits, improper techniques can lead to injuries that affect quality of life and healthcare costs. Therefore, understanding the risk factors associated with these injuries is crucial for mitigating their negative effects (Ceyssens et al., 2019), as such injuries can compel individuals to reduce their physical activity, further burdening healthcare systems.

Running-related injuries (RRIs) are common and include conditions such as runner’s knee, Achilles tendonitis, stress fractures, shin splints, muscle strains, ankle sprains, plantar fasciitis and iliotibial band syndrome (Kakouris et al., 2021). Estimates indicate that over 40.2% of RRIs are associated with foot and ankle mechanics, with more than a third linked to abnormal joint movements (Kakouris et al., 2021). One such abnormality that significantly contributes to RRIs is excessive ankle eversion. When the eversion angle is excessively high, it leads to an over-pronation of the foot. In the stance phase, this excessive eversion and foot pronation cause an internal rotation of the tibia, femur, and pelvis. This alters the kinematics of the lower limb, creating compensatory movements in adjacent joints, which can strain the musculoskeletal system and increase the risk of injury. For instance, excessive ankle eversion (Stacoff et al., 2000; Clement et al., 1981; Nigg and Morlock, 1987; van Mechelen, 1992) during mid-stance generates substantial strain on the medial fibers of the Achilles tendon thereby elevating the risk of developing Achilles tendinopathy (Lorimer and Hume, 2014; Ryan et al., 2009). Moreover, excessive eversion during the stance phase can overload critical muscles such as the flexor digitorum brevis, tibialis posterior, and soleus, potentially leading to conditions like medial tibial stress syndrome (Beck and Osternig, 1994; Becker et al., 2018; Franklyn and Oakes, 2015; Reinking et al., 2017). Various studies have explored lower limb kinematics concerning these injuries, often comparing kinematic parameters between injured and non-injured groups (Ryan et al., 2009; Ferber et al., 2010; Grau et al., 2011; Coventry et al., 2006; Latorre-Román et al., 2019; Ye et al., 2024; Fukuchi et al., 2017; Hein and Grau, 2014; Donoghue et al., 2008). Studies by Donoghue et al. (2008) and Ryan et al. (2009), have identified significant differences in eversion angles between these groups, suggesting a possible correlation between eversion and injury risk.

The etiology of running-related injuries (RRIs) is frequently associated with suboptimal conditioning, excessive training loads, or elevated fatigue levels, which collectively compromise the body’s capacity to absorb and dissipate impact forces. Variability in loading rates and the presence of high-frequency components within ground reaction forces are critical factors influencing injury risk; specifically, elevated loading rates correlate with increased impact forces, while high-frequency components may signify underlying biomechanical stress (Wang et al., 2023). For instance, loading rates that exceed 80 body weights per second (BW·s^−1^) have been associated with a heightened risk of injury among runners, (Yang et al., 2024), while heel strikes are known to generate significant impact forces that, when subjected to repetitive application, may contribute to an increased likelihood of injury over time (Senapati et al., 2024). Jones et al. (2017) On the other hand, several kinematic studies have also shown that changes in kinematics due to fatigue from prolonged running can contribute significantly to the development of RRI (Derrick et al., 2002; Koblbauer et al., 2014) Research by Cheung and Ng (Cheung and Ng, 2007) shows that fatigue significantly alters movement characteristics by limiting physical abilities, making it a critical factor in movement-related injuries. In addition to fatigue, running biomechanics are influenced by different factors such as running speed and incline. As speed increases, joint kinematics and muscle activation patterns change, thereby modifying the biomechanical load on the lower limbs. For instance, faster-running correlates with increased joint flexion and more dynamic movement patterns, affecting the distribution of forces throughout the body (Zandbergen et al., 2023). The duty factor—referring to the proportion of the gait cycle during which the foot is in contact with the ground—decreases at higher speeds, limiting the period available for absorbing impact forces and potentially elevating the risk of injury (Van Hooren et al., 2024). Moreover, Jacobs and Berson (Jacobs and Berson, 1986) found that an increase in training speed is directly correlated with injury. While some other studies found no correlation between training speed and the risk of sustaining injury (van Gent et al., 2007; Rauh et al., 2006). Incline running also significantly affects biomechanics, with uphill running increasing the load on the tibia and placing additional strain on lower leg muscles, raising the risk of tibial stress injuries (Rice et al., 2024). However, incline running has been shown to reduce loading rates and peak vertical ground reaction forces, particularly when speed is adjusted to maintain iso-efficiency, as demonstrated by Williams et al. (2020) Both speed and incline alter spatiotemporal parameters, such as stride length and cadence. Given the substantial influence of these factors on running kinematics, it is crucial to explore how they contribute to the incidence of RRIs. In this context, we aim to monitor the eversion angle of the foot under varying conditions—speed, incline, and fatigue.

Given the complex nature of running biomechanics and the various factors influencing injury risk, it is essential to accurately assess and analyze lower limb kinematics. Kinematic analysis of the lower limbs can be conducted using several methods, with the high-speed dual fluoroscopic imaging system (DFIS) (Ye et al., 2024) and 3D motion capture systems utilizing skeletal markers being among the most accurate (Stacoff et al., 2000). However, these methods are either invasive (Stacoff et al., 2000) or potentially harmful to participants due to X-ray exposure (Ye et al., 2024) making them unsuitable for widespread application. Another slightly less accurate but non-invasive method is the gold standard 3D motion capture with reflective skin markers, allowing for the quantification of angles between body segments such as the calcaneus and tibia (Novacheck, 1998). Several studies have been published using 3D motion capture systems to investigate lower limb kinematics. However, numerous studies have attached reflective markers to the running shoe (Ferber et al., 2010; Fukuchi et al., 2017; Napier et al., 2019), which may lead to inaccurate measurements of subtalar joint movement. To our knowledge, there has been one study that used custom-made shoes that allowed researchers to position markers directly on the skin; however, they investigated the effect of barefoot running (Hein and Grau, 2014).

Present study aims to further explore lower limb kinematics, particularly examining the eversion angle of the ankle joint, which is critical in the development of RRIs. Specifically, we will investigate how different running speeds, inclines, and fatiguing affect the calcaneus angle in injury-free female runners with a heel-to-toe heel strike pattern. By systematically altering running conditions, we seek to identify biomechanical changes that may heighten susceptibility to RRIs.

## 2 Materials and methods

### 2.1 Participants

The study involved 15 active female runners with an average age of 28 ± 13 years, an average body mass of 60 ± 6 kg, and an average height of 168 ± 4 cm. Participants were recruited from the Faculty of Sport and local sports clubs as they were willing to participate. They completed a questionnaire to assess their eligibility based on criteria such as level of physical activity, absence of neurological or chronic non-communicable diseases, and fitness requirements relevant to the study. Importantly, the questionnaire included questions on heel strike foot pattern, an important inclusion criterion. All participants confirmed the heel strike pattern, were physically active for at least 5 h per week, had no neurological or chronic non-communicable diseases, and gave written informed consent prior to inclusion in the study. The heel strike foot pattern was verified prior to the measurements by analysing the participants' heel strike patterns with a high-speed camera. The study was approved by the Committee for Ethical Issues in Sports at the University of Ljubljana, Slovenia (1/2023).

### 2.2 Study design

The participants visited the laboratory once, where an inclusion measurement was first carried out. The main criterion for inclusion in the study was the assumption of a rearfoot running technique. To confirm this, each participant’s heel strike pattern was analysed using a high-speed camera before measurements began. Participants performed short treadmill runs at speeds of 10 and 14 km/h with and without incline.

After the inclusion measurements, reflective markers were attached to the participants, who wore custom running shoes. Additionally, the resting heart rate (HRmin) of the participants was measured, while the maximal heart rate was determined using the well-known formula: HRmax = 220 – age.

#### 2.2.1 Pre-fatigue protocol

**FIGURE 1:**
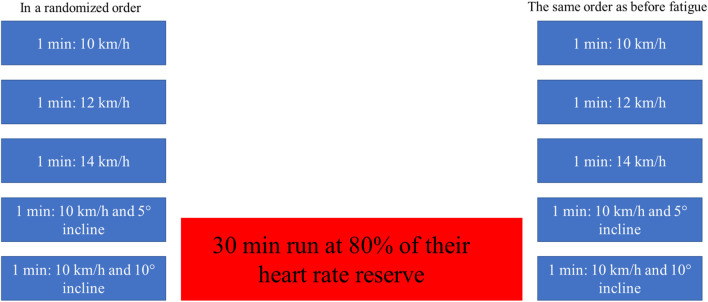
Experimental protocol.

Participants completed two sets of five 1-min treadmill runs under varying conditions to analyze the effects of speed and incline on calcaneus kinematics. The first set (pre-fatigue) included trials at the following conditions: 10 km/h with no incline, 12 km/h with no incline, 14 km/h with no incline, 10 km/h with a 5° incline, and 10 km/h with a 10° incline. Each trial was followed by a 1-min rest (Figure 1). The order of trials was randomized to minimize any order effects. Participants were instructed to set the treadmill to the appropriate speed and incline before each run. After each run, there was a 1-min break.

#### 2.2.2 Fatigue protocol

Following the pre-fatigue test protocol, participants underwent a fatigue phase that consisted of a 30-min run on a flat (no incline) at an intensity corresponding 80% of their heart rate reserve (HRR) (Figure 1). The target heart rate was calculated prior to the start of the measurement using the formula: HRR = [(HRmax–HRmin) × 0.8] + HRmin, where HRmin was resting heart rate. During the fatigue phase, participants self-regulated their running speed to maintain this target heart rate, aided by a heart rate monitor connected to the treadmill. After the fatigue phase, participants self-reported their fatigue level using a visual analog scale (VAS), where 0 indicated no fatigue and 10 indicated extreme fatigue. Thirteen out of fifteen participants reported a fatigue score of 6.4 ± 0.7, corresponding to a threshold between moderate and severe fatigue.

#### 2.2.3 Post-fatigue protocol

After the fatigue run, participants repeated the same five 1-min trials (post-fatigue) under identical conditions as the pre-fatigue test protocol (Figure 1). The aim was to compare biomechanical differences in calcaneus kinematics before and after fatigue across the five test conditions.

Heart rate was measured only during the fatigue protocol, as the primary focus of the study was on ankle kinematics during the 5-run protocol. The short duration of the runs minimized any potential impact of cardiovascular stress on the kinematic results. Furthermore, VAS scores following the repeated 5-run protocol (6.1 ± 1.1) indicate that the short runs did not significantly affect participant fatigue. This is supported by the lack of a statistically significant difference between VAS scores after the fatigue protocol and after the 5-run protocol (*p* = 0.34).

### 2.3 Monitoring

#### 2.3.1 Heel strike foot pattern

A DS-CAM-1100m high-frequency camera set at 300fps, an 8-channel 24 bit and 200 kHz Dewe 43 DAQa with DewesoftX software environment (all DEWESoft, Slovenia) were used to analyse running technique, excluding subjects who did not run over the heel.

#### 2.3.2 Running

**FIGURE 2:**
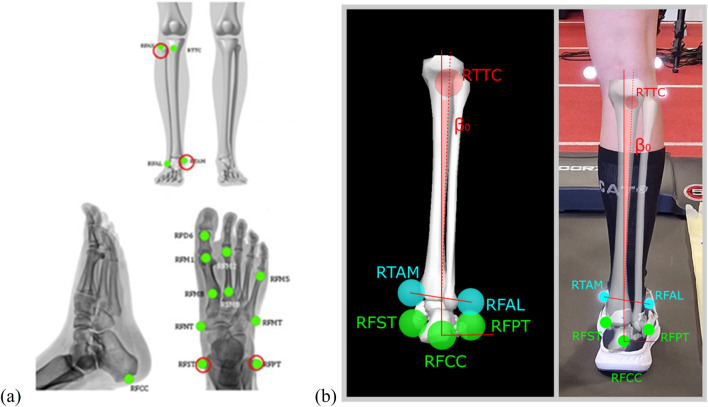
**(A)** Placement of “IORFoot Model” passive markers, **(B)** passive markers and presentation of eversion angle.

The running protocol was performed on a TRX Marathon treadmill (Toorx, Pozzolo Formigaro, Italy) with a belt size of 530 × 1520 mm. The treadmill allowed adjustable speeds from 0.8 to 22.0 km/h and an incline from 0% to 13%. The kinematic data acquisition was performed with a Qualisys Oqus system which consists of 12 infrared cameras (Qualisys AB, Gothenburg, Sweden) operating at a recording frequency of 180 Hz. Reflective markers, each with a diameter of 14 mm (Qualisys AB, Gothenburg, Sweden), were used for the measurements. A total of 43 reflective markers were attached to the body prior to the measurement. Two marker sets were utilized: the Qualisys Sports Marker Set and the IORfoot Marker Set for the foot and lower limbs. From the Qualysis Marker Set we used all the markers while from the IORfoot Marker Set we used only 4 markers that are marked with red circles in Figure 2A. In addition, 4 passive markers were attached to a treadmill in order to calibrate the treadmill in a laboratory. The heart rate was monitored with a Polar H10 device (Polar Electro Oy, Kempele, Finland).

### 2.4 Running shoes

**FIGURE 3:**
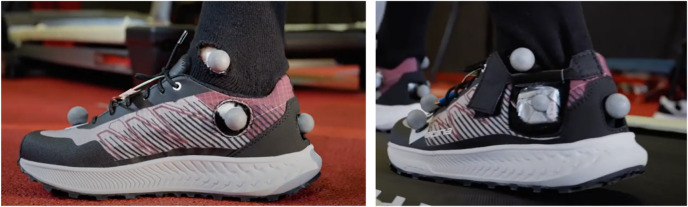
Adapted running shoes with modifications for attaching markers, with openings under the medial malleoli and the heel.

To obtain accurate measurements of the calcaneus angle, the markers must be placed directly on the skin and not on the shoe. Therefore, our running shoes have been specially modified to make this possible. In collaboration with Alpina (Alpina, Žiri, Slovenia), the shoes were redesigned to have small openings in precise locations—under the medial malleolus and around the calcaneus area—to allow the passive reflective markers to be placed directly on the runner’s skin, Figure 3. These strategic openings ensured that the movement of the calcaneus could be accurately measured while still enabling good fixation and comfort of the running shoes. This was a crucial factor in the methodology of the present study to observe the natural calcaneus movement without the interference of the shoe material. Additionally, corresponding openings were created in the socks to ensure that the markers were securely attached directly to the skin.

### 2.5 Data analysis

The kinematic data on the movement of the calcaneus were recorded using the Qualisys kinematic system. The associated Track Manager software enabled the creation of a model for the automatic detection and data acquisition of passive markers. This model enabled the recognition of marker positions in space and the subsequent creation of movement models for body segments. The orientation of the rigid body segments in space was determined by calculating the Euler angles using Visual 3D software (C-Motion, Maryland, United States). Rotations around the anterior-posterior axis (x-axis), specifically the calcaneus inversion/eversion angle, were crucial for the subsequent analysis. Following the guidelines of the International Society of Biomechanics (ISB), (Leardini et al., 2021), a static measurement was performed to establish a neutral position in which the eversion angle was set to 0° for each subject.

The data were then processed in the MATLAB R2021b (Mathworks, Massachusetts, United States) where algorithms recognised and differentiated steps based on the height of the heel markers and calcaneus angles calculated from Visual3D software. Foot contact with the ground was identified by analyzing the local maxima of the inversion angle prior to the minimum z-position of the heel marker, which also needed to be below a threshold value of 3.5 cm. The toe-off event was defined as a 3.5 cm ascent above the minimum z-position of the marker placed on the second toe. During ground contact, we assessed the eversion/inversion angle of calcaneus at heel strike (*ß*_0_), maximum eversion angle of calcaneus (*ß*_MAX_), and calcaneus range of motion–difference between subsequent parameters (*ß*_ROM_) for subsequent statistical analysis. Eversion angle is presented on Figure 2B.

Steps included in the analysis were selected from the end of the 1-min interval. To assess the reliability of the eversion angle measurements, we determined that an analysis of the final 10 s of the running trials, which typically corresponds to around 15 steps, provides a reliable estimate of the eversion angle with a SEM of 0.15°. This duration was chosen because by this point, participants had adapted to the running speed, minimizing variability from initial adjustments.

### 2.6 Statistical analysis

Statistical analyses were conducted using RStudio (Massachusetts, United States). Mixed models were employed to analyze the relationship between the selected response variables and the predictor variables. High inter-subject variability was observed in the sample, as indicated by the residual plots, which revealed heteroscedasticity. To address this, a random effect for individual subjects was included in the models. Initially, we assessed the assumptions of homoscedasticity and normality by examining the residual plots. The assumption of homoscedasticity was violated for all variables. This issue was resolved by incorporating a random subject effect at the intercept in the mixed models, which successfully addressed the violation and ensured compliance with the assumption of homoscedasticity.

Additionaly, standard error of the measurement (SEM) was calculated for all three parameters, i.e., eversion/iversion angle at heel strike, maximal eversion angle, and range of motion. Furthermore, we applied random noise of 1 mm uniformly across all three spatial coordinates to the original marker position data and subsequently reprocessed the data using the Visual3D pipeline to evaluate the effect of the 3D kinematic system error on the derived parameters.

## 3 Results

### 3.1 Influence of different speeds

**FIGURE 4:**
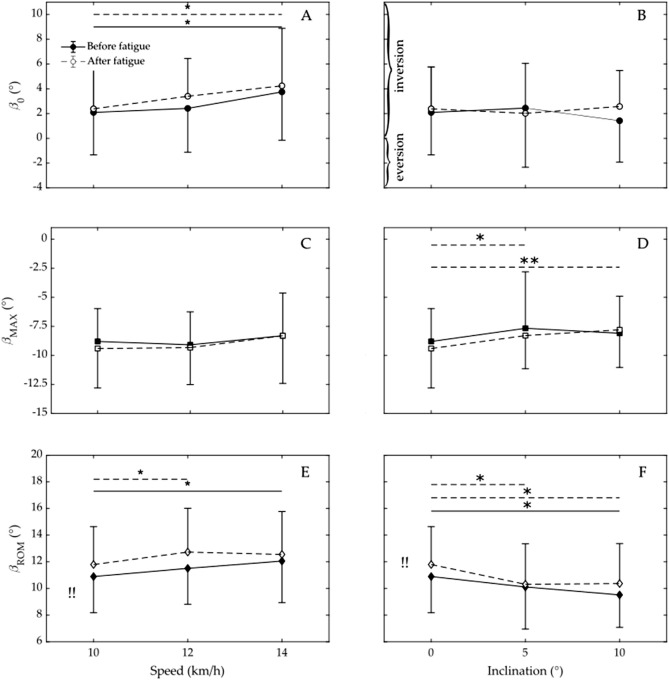
Calcaneus eversion/inversion angle at heel strike (*ß*_0_) across varying speeds **(A)** and inclinations **(B)**; maximum eversion angle ((*ß*_MAX_) at different speeds **(C)** and inclinations **(D)**; and range of motion in the calcaneus (*ß*_ROM_) for varying speeds **(E)** and inclinations **(F)**. Data prior to the onset of fatigue is indicated by filled circles (squares, diamonds) and a solid line, whereas data subsequent to fatigue is represented by open circles (squares, diamonds) and a dashed line. * Indicates significant difference (*p* < 0.05), ** Indicates highly significant difference (*p* < 0.001), !! Indicates that fatigue has an overall significant effect on *ß*_ROM_ (*p* < 0.05).

Before fatigue (Figure 4; Table 1), variable speed had no statistically significant overall effect on *ß*_0_ *p* = 0.07). However, *ß*_0_ was significantly higher during running at 14 km/h compared to running at 10 km/h (1.8° ± 3.9° vs. 2.1° ± 3.4°, *p* = 0.03). There was no significant difference in *ß*_0_ between running at 12 km/h and 10 km/h (*p* = 0.65). In contrast, variable speed after fatigue (Table 2) had a statistically significant overall effect on *ß*_0_ (*p* = 0.05). In addition, *ß*_0_ was significantly higher when running at 14 km/h (3.4° ± 3.0° vs. 2.4° ± 3.4°, *p* = 0.17), but not when running at 12 km/h compared to 10 km/h (4.2° ± 4.6° vs. 2.4° ± 3.4°, *p* = 0.01).

**TABLE 1:** Three-dimensional kinematics of the lower leg during running prior to the onset of fatigue. Presented are the group means ± standard deviation (SD).

Before fatigue	10 km/h	12 km/h	14 km/h	10 km/h, 5° incline	10 km/h, 10° incline
*ß*_0_ [°]	2.1 ± 3.4	2.4 ± 3.5	3.8 ± 3.9*	2.4 ± 4.8	1.4 ± 3.3
*ß*_MAX_ [°]	−8.8 ± 2.8	−9.1 ± 2.8	−8.3 ± 3.7	−7.7 ± 4.9	−8.1 ± 3.2
*ß*_ROM_ [°]	10.9 ± 2.7	11.5 ± 2.7	12.1 ± 3.1*	10.1 ± 3.2	9.5 ± 2.4†

*Statistically significant compared to 10 km/h (*p* < 0.05), †Statistically significant compared to 0° inclination (*p* < 0.05).

**TABLE 2:** Three-dimensional kinematic analysis of the lower leg during running post-fatigue. The results are presented as group means ± standard deviation (SD).

After fatigue	10 km/h	12 km/h	14 km/h	10 km/h, 5° incline	10 km/h, 10° incline
*ß*_0_ [°]	2.4 ± 3.4	3.4 ± 3.0	4.2 ± 4.6*	2.1 ± 4.0	2.6 ± 2.9
*ß*_MAX_ [°]	−9.4 ± 3.4	−9.3 ± 3.2	−8.3 ± 4.1	−8.3 ± 2.9†	−7.8 ± 3.2††
*ß*_ROM_ [°]	11.8 ± 2.8	12.7 ± 3.3*	12.6 ± 3.2	10.3 ± 3.0†	10.4 ± 3.0†

*Statistically significant compared to 10 km/h (*p* < 0.05), †Statistically significant compared to 0° inclination (*p* < 0.05), ††Statistically significant compared to 0° inclination (*p* < 0.001).

Variable speed had no statistically significant overall effect on *ß*_MAX_ before (*p* = 0.47, Table 1)or after fatigue (*p* = 0.25, Table 2). There was also no statistically significant difference in *ß*_MAX_ when running at 12 or 14 km/h compared to 10 km/h, either before (*p* = 0.66 and *p* = 0.44, respectively) or after fatigue (*p* = 0.91 and *p* = 0.13, respectively).

Variable speed had a statistically significant overall effect on *ß*_ROM_ before (*p* = 0.02, Table 1) but not after fatigue (*p* = 0.09, Table 2). In addition, *ß*_ROM_ was statistically significant when running at 14 km/h before fatigue and when running at 12 km/h after fatigue compared to 10 km/h (10.9° ± 2.7° vs. 12.1 ± 3.1, *p* = 0.004, and 11.8 ± 2.8 vs. 12.7 ± 3.3*, *p* = 0.04, respectively).

### 3.2 Influence of different inclinations

Before and after fatigue (Figure 4; Tables 1, 2), the variable inclination had no statistically significant overall effect on *ß*_0_ (*p* = 0.57 and *p* = 0.52, respectively). Before fatigue (Table 1), *ß*_0_ during running at 5° or 10° also did not differ significantly from that with no incline (*p* = 0.68 and 0.08, respectively). Even after fatigue (Table 2), *ß*_0_ during running with an inclination of 5° or 10° did not differ significantly from that with no incline (*p* = 0.46 and 0.70, respectively).

Before fatigue (Table 1), the inclination had no overall statistically significant effect on *ß*_MAX_ (*p* = 0.42). After fatigue (Table 2), however, the inclination had a statistically significant effect on *ß*_MAX_ (*p* = 0.002). In addition, *ß*_MAX_ was significantly lower when running at 5° or 10° than with no incline (−9.4° ± 3.4° vs. −8.3° ± 2.9°, *p* = 0.01, and −9.4° ± 3.4° vs. −7.8° ± 3.2°, *p* = 0.0003).

Before and after fatigue (Tables 1, 2), inclination had an overall statistically significant effect on *ß*_ROM_ (*p* = 0.02, and 0.003, respectively). In addition, *ß*_ROM_ was significantly lower when running with a 10° incline before or after fatigue compared to no incline (9.5° ± 2.4° vs. 10.9° ± 2.7°, *p* = 0.004 and 10.4° ± 3.0° vs. 11.8° ± 2.8°, *p* = 0.003, respectively). Moreover *ß*_ROM_ after fatigue was also significantly different when running with a 5° incline compared to running with no incline (10.3° ± 3.0° vs. 11.8° ± 2.8°, *p* = 0.002).

### 3.3 Influence of fatigue

Overall, fatigue (Table 2) had no significant effect on *ß*_0_ (*p* = 0.70). Furthermore, fatigue did not have a significantly different effect on *ß*_0_ at speed of 12 km/h or 14 km/h compared to 10 km/h (*p* = 0.52 and *p* = 0.85, respectively). Moreover, fatigue did not have a significantly different effect on *ß*_0_ at incline of 5° or 10° compared to no incline (*p* = 0.50 and *p* = 0.41, respectively).

Fatigue (Table 2) had no significant overall effect on *ß*_MAX_ (*p* = 0.38). Furthermore, fatigue did not have a significantly different effect on *ß*_MAX_ at speed of 12 km/h or 14 km/h compared to 10 km/h (*p* = 0.71 and *p* = 0.33, respectively). Moreover, fatigue did not have a significantly different effect on *ß*_MAX_ at incline of 5° or 10° compared to no incline (*p* = 0.54 and *p* = 0.99, respectively).

Fatigue (Table 2) had an overall significant effect on *ß*_ROM_ (*p* = 0.05). However, fatigue did not have a significantly different effect on *ß*_ROM_ at speed of 12 km/h or 14 km/h compared to 10 km/h (*p* = 0.59 and *p* = 0.50, respectively). Moreover, fatigue did not have a significantly different effect on *ß*_MAX_ at incline of 5° or 10° compared to no incline (*p* = 0.27 and *p* = 0.94, respectively).

### 3.4 Reliability and accuracy of the measurements

Standard error of measurement (SEM) for *ß*_0_, *ß*_MAX_ and *ß*_ROM_ were calculated to be 0.08°, 0.06°, and 0.05° respectively. Furthermore, we assessed the effect of random noise introduced into the data, and our findings showed that the SEM increased slightly, with values of 0.09°, 0.06°, and 0.06°. Additionaly, the variability of these angles was 0.7°, 0.6°, and 0.5°.

## 4 Discussion

The main aims of the present study were to determine whether different speeds, inclinations, or fatigue affected the calcaneus eversion/inversion angle at heel strike, the maximum eversion angle, or the range of motion of the calcaneus inversion/eversion angle. Findings in the present study indicate that speed significantly influenced the eversion/inversion angle at heel strike when participants were fatigued. Inclination had a statistically significant effect on maximum eversion angle after fatigue and on range of motion both before and after fatigue. Additionally, fatigue itself had a significant impact on *ß*_ROM_.

The reliability of the measured angles is indicated by the low Standard Error of the Mean (SEM) values, which are below 0.1°. The accuracy of the measurements was assessed by calculating the SEM while applying a uniform random noise of 1 mm across all three spatial coordinates to the original marker position data. The data were then reprocessed using the Visual3D pipeline to evaluate the impact of 3D kinematic system error on the derived parameters. The SEM exhibited a slight increase compared to the SEM calculated without the additional noise. However, this increase was minimal, indicating that the measurement error associated with the introduced noise did not significantly compromise the accuracy of the measurements. Therefore, we can conclude that the measurement system maintains a high level of accuracy, even in the presence of noise. All previously mentioned parameters are important regarding the prevention of RRI. As noted in prior publications *ß*_0_ has been linked to RRIs, including iliotibial band syndrome (Grau et al., 2011) and Achilles tendinopathy. (McCRORY et al., 1999; Mousavi et al., 2019). However, existing literature comparing injured and non-injured runner groups presents conflicting results. Some studies indicate that uninjured runners exhibit a greater *ß*_0_ (Kuhman et al., 2016; Hreljac et al., 2000), while other studies suggest the opposite (Grau et al., 2008) or report no significant difference (Hamacher et al., 2016). Additionally, *ß*_MAX_ can increase strain on the Achilles tendon and other related structures, potentially leading to conditions such as medial tibial stress syndrome. (Stacoff et al., 2000; Ye et al., 2024). Willems et al. (2006) showed that an increased *ß*_MAX_ raises the risk of exercise-related lower leg pain. In their study, *ß*_MAX_ was significantly higher in injured participants (9.6° ± 5.9°) compared to uninjured participants (7.7° ± 5.1°), with the difference being statistically significant (*p* = 0.03). Furthermore, *ß*_ROM_ is correlated with chronic ankle instability (CAI) (Cordova et al., 2000; Stotz et al., 2021). Individuals with a higher *ß*_ROM_ may therefore be at an increased risk, suggesting that a greater *ß*_ROM_ could predict a higher likelihood of RRI.

In the current study, the pre-fatigue and after-fatigue values of *ß*_0_ (ranging from 2.1° to 3.4° and 2.4°–4.2°) are consistent with values reported in previous research: 2.7° for the left leg and 2.5° for the right leg by Goetze (2015), 4° by Hamacher et al. (2016), 3.5° by Stacoff et al. (2000) In all of the mentioned studies, markers were placed directly on the shoes rather than on the skin; however, as a previous study demonstrated (Sinclair et al., 2013) this should not have affect the values of *ß*_0_, although it does have a significant impact on *ß*_MAX_ and consequently on *ß*_ROM_. As mentioned above Willems et al. (2006) measured *ß*_MAX_ in injured participants at 9.6° ± 5.9° and 7.7° ± 5.1° in unijured participants. In the current study, *ß*_MAX_ in uninjured participants ranged between 8.3° and 9.4° when running on a flat surface The slight discrepancy in these values could be attributed to the participants in the present study running in footwear, while those in the previously mentioned study ran barefoot; this is in agreement with a previous study by Thompson et al. (2015). This finding is consistent with the results of Stacoff et al. (2000) who used intracortical Hofmann pins to measure *ß*_MAX_ in barefoot and shod running, *ß*_MAX_ of 6.9° ± 0.7° and 8.8° ± 1.5°, respectively. Therefore, we can confirm that values in the present study align with the literature for uninjured runners. The *ß*_ROM_ observed in the present study is comparable to that reported by Hein and Grau, (Hein and Grau, 2014), who measured *ß*_ROM_ while participants ran either in shoes or barefoot at a speed of 11 km/h. They found a *ß*_ROM_ of 12° ± 3° when running in shoes, which is similar to the 10.9° ± 2.7° and 11.5° ± 2.7° *ß*_ROM_ we observed at speeds of 10 and 12 km/h, respectively.

### 4.1 Influence of different speeds on *ß*_0,_ *ß*_MAX,_ and *ß*_ROM_

In the current study, running at 14 km/h led to a significantly higher *ß*_0_ compared to running at 10 km/h, both prior to and following fatigue. A higher *ß*_0_ could be attributed to a greater proportion of foot placement on the middle and front foot as running speed increases (Keller et al., 1996). This is consitetn with the results published by Koldenhoven et al. (2019) observed across 3 walking speeds—preferred walking speed, 120% of preferred walking speed, and a standardized faster speed. Their findings indicated that participants with chronic ankle instability displayed greater inversion at higher speeds, while those who had previously experienced an ankle sprain but had returned to pre-injury function demonstrated increased eversion at greater speeds.

Contrary, there was no statistically significant difference in *ß*_MAX_ when participants ran at different speeds. Although there was a noticeable trend indicating a smaller *ß*_MAX_ when running at 14 km/h compared to 10 km/h, both in fatigued and non-fatigued states, this trend was not statistically significant. This finding is consistent with the study by Koldenhoven et al. (2019), Conversely, another study indicated an increased *ß*_MAX_ with increased speed; (Fukuchi et al., 2017); however, that study placed markers directly on the running shoes, thus measuring the eversion of the foot rather than that of the calcaneus. As mentioned above, *ß*_MAX_ is significantly different when markers are placed on the shoes compared to when they are placed directly on the skin (Sinclair et al., 2013).

Moreover, speed had a significant overall effect on *ß*_ROM_ in the non-fatigued state, with higher speed leading to an increased range of motion. This finding aligns with the results from Fukuchi et al. (2017), who examined running at three different speeds (9, 12.6, and 16.2 km/h) and observed that higher speeds were associated with an increased range of motion. However, in the fatigued state in our study, only running at 12 km/h resulted in a significant increase in *ß*_ROM_ compared to running at 10 km/h.

As previously discussed, an increased *ß*_ROM_ is associated with CAI, which is a known predictor of RRI. Furthermore, although we observed a trend toward a higher *ß*_MAX_ with increased running speed, this trend did not reach statistical significance. Nevertheless, these findings suggest that higher running speeds may elevate the risk of RRIs, warranting caution and further investigation.

### 4.2 Influence of different inclinations on *ß*_0,_ *ß*_MAX,_ and *ß*_ROM_

In the present research, we found that inclination did not significantly affect *ß*_0_, which aligns with the findings of Sinclair et al. (2018) Additionally, *ß*_0_ before fatigue we observed at inclinations of 0°, 5°, and 10° are comparable to those reported in the previous study (2.9° vs. 2.1°, 3.7° vs. 2.4°, and 2.2° vs. 1.4°, respectively) (Sinclair et al., 2018). However, Dixon et al. (2011) reported a higher *ß*_0_ when running uphill at a 10° incline. The key difference between Dixon et al.’s study and the studies conducted by Sinclair and the present study is that the participants in Dixon’s study were running barefoot, whereas in both Sinclair’s study and the current, the participants were running in shoes. Therefore, we can conclude that uphill running in running shoes does not significantly alter the inversion angle at foot strike.

In the non-fatigued state, inclination had no statistically significant effect on *ß*_MAX_. However, when participants were fatigued, *ß*_MAX_ was lower when running uphill at either 5° or 10°. This suggests that running uphill in a fatigued state may reduce the risk of injury due to excessive calcaneal eversion. This finding for non-fatigued state is consistent with that of Sinclair et al. (2018), who found no statistically significant difference in *ß*_MAX_ at different inclinations.

Additionally, we can speculate that the development of RRI may be reduced due to smaller *ß*_ROM_ when running at an incline of 10° in both fatigued and non-fatigued states, and at an incline of 5° when in a fatigued state, where the results were statistically significant. However, Sinclair et al. (2018) found no statistically significant difference in this parameter when evaluating different inclines. There was an observable trend, with *ß*_ROM_ recorded at 11.21° ± 5.59° for a 5° incline and 9.89° ± 4.16° for a 15° incline; however, as previously noted, this difference was not statistically significant. The absolute values of *ß*_ROM_ were comaprable in both studies (11.21° ± 5.59° vs. 10.1 ± 3.2 at the 5° incline).

### 4.3 Influence of fatigue on *ß*_0,_ *ß*_MAX,_ and *ß*_ROM_

Fatigue did not affect the *ß*_0_ across different speeds or inclinations in present study. These observations are consistent with a previous study, (Van Gheluwe and Madsen, 1997), which also found that *ß*_0_ remained unchanged before and after fatigue. In that study, markers were placed on the shoes, which, as mentioned earlier, differs from placing markers directly on the skin (Sinclair et al., 2013).

In addition, fatigue did not have a significant effect on *ß*_MAX_ across different speeds or inclinations. Although there was a visible trend toward higher *ß*_MAX_ after fatigue when running at 10 or 12 km/h and at a 5° incline, the differences were not statistically significant. We initially expected that *ß*_MAX_ would change after fatigue, as previous studies have suggested that foot and joint kinematics are altered at key points in the running cycle due to fatigue (Van Gheluwe and Madsen, 1997; Elliott and Roberts, 1980). To our knowledge, only one study has measured *ß*_MAX_ during shod running in fatigued state with markers attached directly to the skin (Brown et al., 2014). However, this study did not examine the effect of fatigue on *ß*_MAX_ but rather compared the dominant and non-dominant limbs before and after fatigue. As a result, it remains unclear whether fatigue has a statistically significant impact on *ß*_MAX_. Nonetheless, their results are similar to present study, showing a change in eversion angle of 1.8° in the dominant limb and 0.6° in the non-dominant limb after fatigue during running at 12 km/h. In present study, the change was slightly smaller, at just 0.2°. Another study (Van Gheluwe and Madsen, 1997) showed that measured *ß*_MAX_ found a statistically significant difference after fatigue, but the markers in that study were placed on the shoes, which does not directly reflect calcaneus motion, as mentioned earlier. It is also possible that participants in the present study, who were required to be active for at least 5 h per week, were well-trained, and the 0.5-h exhaustion run may not have been sufficient to induce significant fatigue.

Fatigue has a substantial impact on *ß*_ROM_, indicating that running while fatigued may elevate the risk of developing RRI. This finding aligns with the research conducted by Koblbauer et al. (2014) which demonstrated that the range of motion in the non-dominant leg was significantly higher in a fatigued state compared to pre-fatigued conditions. While the running speed was not reported in their study, the results are consistent with our findings for running at 12 km/h (11.5° ± 3.9° vs. 11.5° ± 2.7° in the non-fatigued and 13.1° ± 4.6° vs. 12.7° ± 3.3° in the fatigued state).

## 5 Conclusion

This study aimed to explore the impact of different running speeds, inclines, and levels of fatigue on the calcaneus eversion/inversion angle at heel strike, maximum eversion angle and range of motion during running in injury-free female runners. The findings indicate that speed significantly influences *ß*_0_, particularly when participants are fatigued. Inclination also had a statistically significant effect on *ß*_MAX_ after fatigue and *ß*_ROM_ both before and after fatigue. Additionally, fatigue itself was found to significantly impact the calcaneus *ß*_ROM_, The findings suggest that running at higher speeds and in a fatigued state increases the likelihood of RRI due to the higher *ß*_ROM_, while running at an incline may reduce this risk by lowering the *ß*_ROM_ as well as *ß*_MAX_. These results underscore the importance of considering speed, incline, and fatigue in injury prevention strategies for runners with heel-to-toe foot strike. However, it is important to note that these conclusions are specific to how the kinematics of the calcaneus can influence the risk of RRI.

## 6 Limitations

The findings of the study should be interpreted with some caution due to its limitations. Firstly, the sample size was relatively small, comprising only 15 female participants. This restricts the generalizability of the results to a wider population, including male runners. The decision to focus on female runners was based on observed differences in injury prevalence and biomechanics between genders, particularly in lower limb injuries related to running (Rubio et al., 2023; Xie et al., 2022). However, future studies should consider including male participants to determine whether the findings are consistent across genders. Secondly, this study focused only on injury-free participants, which may not fully capture the variability in running biomechanics that is seen in individuals with a history of running-related injuries (RRIs). It is well known that individuals with previous injuries often demonstrate altered running patterns and altered kinematics, which could impact the results. While the inclusion of injury-free participants helps standardize the biomechanical measurements, the results may not fully reflect the injury risk for those with past injuries. Additionaly, the study exclusively included runners with a heel-to-toe foot strike pattern. While this is common among distance runners, it does not account for the biomechanics of runners who adopt a midfoot or forefoot strike. Different foot strike patterns could result in different kinematic patterns, and thus, future studies should examine whether the observed findings are consistent across a variety of foot strike patterns. Furthemore, the fatigue protocol involved a 30-min run, which may not have resulted in the same level of fatigue as longer or more intense running sessions. Further research with a larger and more diverse sample, as well as varying fatigue protocols, is necessary to validate and expand upon these findings. The study was conducted in a controlled laboratory setting, which may not fully replicate the conditions encountered by runners in real-world environment. Finally, the use of reflective markers to capture kinematic data can also present limitations, despite efforts to minimize errors by affixing markers directly to the skin within the customized running shoes. Although this approach reduces errors commonly associated with marker placement on shoes, there can still be inaccuracies in attaching the markers to anatomical landmarks despite a low standard error was observed in the studied parameters.

## Data Availability

The raw data supporting the conclusions of this article will be made available by the authors, without undue reservation.

## References

[B1] Beck B. R. Osternig L. R. (1994). Medial tibial stress syndrome. The location of muscles in the leg in relation to symptoms. J. Bone Jt. Surg. Am. 76, 1057–1061. 10.2106/00004623-199407000-00015 8027114

[B2] Becker J. Nakajima M. Wu W. F. W. (2018). Factors contributing to medial tibial stress syndrome in runners: a prospective study. Med. Sci. Sports Exerc 50, 2092–2100. 10.1249/MSS.0000000000001674 29787473

[B3] Brown A. M. Zifchock R. A. Hillstrom H. J. (2014). The effects of limb dominance and fatigue on running biomechanics. Gait Posture 39, 915–919. 10.1016/j.gaitpost.2013.12.007 24405748

[B4] Ceyssens L. Vanelderen R. Barton C. Malliaras P. Dingenen B. (2019). Biomechanical risk factors associated with running-related injuries: a systematic review. Sports Med. Auckl N. Z. 49, 1095–1115. 10.1007/s40279-019-01110-z 31028658

[B5] Cheung R. T. H. Ng G. Y. F. (2007). Efficacy of motion control shoes for reducing excessive rearfoot motion in fatigued runners. Phys. Ther. Sport 8, 75–81. 10.1016/j.ptsp.2006.12.002

[B6] Clement D. B. Taunton J. E. Smart G. W. McNicol K. L. (1981). A survey of overuse running injuries. Phys. Sportsmed. 9, 47–58. 10.1080/00913847.1981.11711077 27453020

[B7] Cordova M. L. Ingersoll C. D. LeBlanc M. J. (2000). Influence of ankle support on joint range of motion before and after exercise: a meta-analysis. J. Orthop. Sports Phys. Ther. 30, 170–177. 10.2519/jospt.2000.30.4.170 10778794

[B8] Coventry E. O’Connor K. M. Hart B. A. Earl J. E. Ebersole K. T. (2006). The effect of lower extremity fatigue on shock attenuation during single-leg landing. Clin. Biomech. Bristol Avon 21, 1090–1097. 10.1016/j.clinbiomech.2006.07.004 16949185

[B9] Derrick T. R. Dereu D. McLean S. P. (2002). Impacts and kinematic adjustments during an exhaustive run. Med. Sci. Sports Exerc 34, 998–1002. 10.1097/00005768-200206000-00015 12048328

[B10] Dixon P. C. Tisseyre M. Damavandi M. Pearsall D. J. (2011). Inter-segment foot kinematics during cross-slope running. Gait Posture 33, 640–644. 10.1016/j.gaitpost.2011.02.010 21420865

[B11] Donoghue O. A. Harrison A. J. Laxton P. Jones R. K. (2008). Lower limb kinematics of subjects with chronic achilles tendon injury during running. Res. Sports Med. Print 16, 23–38. 10.1080/15438620701693231 18373287

[B12] Durstine J. L. Gordon B. Wang Z. Luo X. (2013). Chronic disease and the link to physical activity. J. Sport Health Sci. 2, 3–11. 10.1016/j.jshs.2012.07.009

[B13] Elliott B. C. Roberts A. D. (1980). A biomechanical evaluation of the role of fatigue in middle-distance running. Can. J. Appl. Sport Sci. J. Can. Sci. Appl. Au Sport 5, 203–207. 7449034

[B14] Ferber R. Noehren B. Hamill J. Davis I. S. (2010). Competitive female runners with a history of iliotibial band syndrome demonstrate atypical hip and knee kinematics. J. Orthop. Sports Phys. Ther. 40, 52–58. 10.2519/jospt.2010.3028 20118523

[B15] Franklyn M. Oakes B. (2015). Aetiology and mechanisms of injury in medial tibial stress syndrome: current and future developments. World J. Orthop. 6, 577–589. 10.5312/wjo.v6.i8.577 26396934 PMC4573502

[B16] Fukuchi R. K. Fukuchi C. A. Duarte M. (2017). A public dataset of running biomechanics and the effects of running speed on lower extremity kinematics and kinetics. PeerJ 5, e3298. 10.7717/peerj.3298 28503379 PMC5426356

[B17] Goetze I. (2015). The etiology of running induced overuse injuries: an individual and multifactorial approach. Köln: Deutsche Sporthochschule Köln.

[B18] Grau S. Krauss I. Maiwald C. Axmann D. Horstmann T. Best R. (2011). Kinematic classification of iliotibial band syndrome in runners. Scand. J. Med. Sci. Sports 21, 184–189. 10.1111/j.1600-0838.2009.01045.x 19903313

[B19] Grau S. Maiwald C. Krauss I. Axmann D. Horstmann T. (2008). The influence of matching populations on kinematic and kinetic variables in runners with iliotibial band syndrome. Res. Q. Exerc Sport 79, 450–457. 10.1080/02701367.2008.10599511 19177946

[B20] Hamacher D. Hollander K. Zech A. (2016). Effects of ankle instability on running gait ankle angles and its variability in young adults. Clin. Biomech. Bristol Avon 33, 73–78. 10.1016/j.clinbiomech.2016.02.004 26954892

[B21] Hein T. Grau S. (2014). Can minimal running shoes imitate barefoot heel-toe running patterns? A comparison of lower leg kinematics. J. Sport Health Sci. 3, 67–73. 10.1016/j.jshs.2014.03.002

[B22] Hreljac A. Marshall R. N. Hume P. A. (2000). Evaluation of lower extremity overuse injury potential in runners. Med. Sci. Sports Exerc 32, 1635–1641. 10.1097/00005768-200009000-00018 10994917

[B23] Jacobs S. J. Berson B. L. (1986). Injuries to runners: a study of entrants to a 10,000 meter race. Am. J. Sports Med. 14, 151–155. 10.1177/036354658601400211 3717487

[B24] Jones C. M. Griffiths P. C. Mellalieu S. D. (2017). Training load and fatigue marker associations with injury and illness: a systematic review of longitudinal studies. Sports Med. Auckl N. Z. 47, 943–974. 10.1007/s40279-016-0619-5 PMC5394138 27677917

[B25] Kakouris N. Yener N. Fong D. T. P. (2021). A systematic review of running-related musculoskeletal injuries in runners. J. Sport Health Sci. 10, 513–522. 10.1016/j.jshs.2021.04.001 33862272 PMC8500811

[B26] Keller T. S. Weisberger A. M. Ray J. L. Hasan S. S. Shiavi R. G. Spengler D. M. (1996). Relationship between vertical ground reaction force and speed during walking, slow jogging, and running. Clin. Biomech. Bristol Avon 11, 253–259. 10.1016/0268-0033(95)00068-2 11415629

[B27] Koblbauer I. F. van Schooten K. S. Verhagen E. A. van Dieën J. H. (2014). Kinematic changes during running-induced fatigue and relations with core endurance in novice runners. J. Sci. Med. Sport 17, 419–424. 10.1016/j.jsams.2013.05.013 23790535

[B28] Koldenhoven R. M. Hart J. Saliba S. Abel M. F. Hertel J. (2019). Gait kinematics and kinetics at three walking speeds in individuals with chronic ankle instability and ankle sprain copers. Gait Posture 74, 169–175. 10.1016/j.gaitpost.2019.09.010 31525655

[B29] Kuhman D. J. Paquette M. R. Peel S. A. Melcher D. A. (2016). Comparison of ankle kinematics and ground reaction forces between prospectively injured and uninjured collegiate cross country runners. Hum. Mov. Sci. 47, 9–15. 10.1016/j.humov.2016.01.013 26827155

[B30] Latorre-Román P. A. García-Pinillos F. Soto-Hermoso V. M. Muñoz-Jiménez M. (2019). Effects of 12 weeks of barefoot running on foot strike patterns, inversion–eversion and foot rotation in long-distance runners. J. Sport Health Sci. 8, 579–584. 10.1016/j.jshs.2016.01.004 31720071 PMC6835025

[B31] Leardini A. Stebbins J. Hillstrom H. Caravaggi P. Deschamps K. Arndt A. (2021). ISB recommendations for skin-marker-based multi-segment foot kinematics. J. Biomech. 125, 110581. 10.1016/j.jbiomech.2021.110581 34217032

[B32] Lorimer A. V. Hume P. A. (2014). Achilles tendon injury risk factors associated with running. Sports Med. Auckl N. Z. 44, 1459–1472. 10.1007/s40279-014-0209-3 24898814

[B33] McCRORY J. L. Martin D. F. Lowery R. B. Cannon D. W. Curl W. W. Read H. M. J. (1999). Etiologic factors associated with Achilles tendinitis in runners. Med. Sci. Sports Exerc 31, 1374–1381. 10.1097/00005768-199910000-00003 10527307

[B34] Mousavi S. H. Hijmans J. M. Rajabi R. Diercks R. Zwerver J. van der Worp H. (2019). Kinematic risk factors for lower limb tendinopathy in distance runners: a systematic review and meta-analysis. Gait Posture 69, 13–24. 10.1016/j.gaitpost.2019.01.011 30658311

[B35] Napier C. MacLean C. L. Maurer J. Taunton J. E. Hunt M. A. (2019). Kinematic correlates of kinetic outcomes associated with running-related injury. J. Appl. Biomech. 35, 123–130. 10.1123/jab.2018-0203 30421631

[B36] Nigg B. M. Morlock M. (1987). The influence of lateral heel flare of running shoes on pronation and impact forces. Med. Sci. Sports Exerc 19, 294–302. 10.1249/00005768-198706000-00017 3600244

[B37] Novacheck T. F. (1998). The biomechanics of running. Gait Posture 7, 77–95. 10.1016/s0966-6362(97)00038-6 10200378

[B38] Pedisic Z. Shrestha N. Kovalchik S. Stamatakis E. Liangruenrom N. Grgic J. (2020). Is running associated with a lower risk of all-cause, cardiovascular and cancer mortality, and is the more the better? A systematic review and meta-analysis. Br. J. Sports Med. 54, 898–905. 10.1136/bjsports-2018-100493 31685526

[B39] Rauh M. J. Koepsell T. D. Rivara F. P. Margherita A. J. Rice S. G. (2006). Epidemiology of musculoskeletal injuries among high school cross-country runners. Am. J. Epidemiol. 163, 151–159. 10.1093/aje/kwj022 16306308

[B40] Reinking M. F. Austin T. M. Richter R. R. Krieger M. M. (2017). Medial tibial stress syndrome in active individuals: a systematic review and meta-analysis of risk factors. Sports Health 9, 252–261. 10.1177/1941738116673299 27729482 PMC5435145

[B41] Rice H. Kurz M. Mai P. Robertz L. Bill K. Derrick T. R. (2024). Speed and surface steepness affect internal tibial loading during running. J. Sport Health Sci. 13, 118–124. 10.1016/j.jshs.2023.03.004 36931595 PMC10818105

[B42] Rubio J. E. Tong J. Sundaramurthy A. Subramani A. V. Kote V. B. Baggaley M. (2023). Differences in running biomechanics between young, healthy men and women carrying external loads. Front. Bioeng. Biotechnol. 11, 1250937. 10.3389/fbioe.2023.1250937 37854880 PMC10579583

[B43] Ryan M. Grau S. Krauss I. Maiwald C. Taunton J. Horstmann T. (2009). Kinematic analysis of runners with achilles mid-portion tendinopathy. Foot Ankle Int. 30, 1190–1195. 10.3113/FAI.2009.1190 20003878

[B44] Senapati D. Rao C. L. Pandey M. (2024). “Finite element analysis of impact forces in running: evaluating footwear strike patterns, and load transfer,” in Material strength and applied mechanics (Amsterdam, Netherlands: IOP Press), 546–552. 10.3233/ATDE240592

[B45] Sinclair J. Atkins S. Vincent H. (2018). The effects of various running inclines on three-segment foot mechanics and plantar fascia strain. Hum. Mov. 15, 209–215. 10.1515/humo-2015-0013

[B46] Sinclair J. Greenhalgh A. Taylor P. Edmundson C. Brooks D. Hobbs S. (2013). Differences in tibiocalcaneal kinematics measured with skin- and shoe-mounted markers. Hum. Mov. 14, 64–69. 10.2478/humo-2013-0005

[B47] Stacoff A. Nigg B. M. Reinschmidt C. van den Bogert A. J. Lundberg A. (2000). Tibiocalcaneal kinematics of barefoot versus shod running. J. Biomech. 33, 1387–1395. 10.1016/s0021-9290(00)00116-0 10940397

[B48] Stotz A. John C. Gmachowski J. Rahlf A. L. Hamacher D. Hollander K. (2021). Effects of elastic ankle support on running ankle kinematics in individuals with chronic ankle instability and healthy controls. Gait Posture 87, 149–155. 10.1016/j.gaitpost.2021.04.037 33933933

[B49] Thompson M. A. Lee S. S. Seegmiller J. McGowan C. P. (2015). Kinematic and kinetic comparison of barefoot and shod running in mid/forefoot and rearfoot strike runners. Gait Posture 41, 957–959. 10.1016/j.gaitpost.2015.03.002 25827681

[B50] van Gent R. N. Siem D. van Middelkoop M. van Os A. G. Bierma-Zeinstra S. M. A. Koes B. W. (2007). Incidence and determinants of lower extremity running injuries in long distance runners: a systematic review. Br. J. Sports Med. 41, 469–480. 10.1136/bjsm.2006.033548 17473005 PMC2465455

[B51] Van Gheluwe B. Madsen C. (1997). Frontal rearfoot kinematics in running prior to volitional exhaustion. J. Appl. Biomech. 13, 66–75. 10.1123/jab.13.1.66

[B52] Van Hooren B. Jukic I. Cox M. Frenken K. G. Bautista I. Moore I. S. (2024). The relationship between running biomechanics and running economy: a systematic review and meta-analysis of observational studies. Sports Med. 54, 1269–1316. 10.1007/s40279-024-01997-3 38446400 PMC11127892

[B53] van Mechelen W. (1992). Running injuries. A review of the epidemiological literature. Sports Med. Auckl N. Z. 14, 320–335. 10.2165/00007256-199214050-00004 1439399

[B54] Wang X. Cai W. Ren D. Yuan H. Ruan M. (2023). Time and frequency domain analysis of ground reaction force in highly cushioned shoes with a carbon-fiber plate. Footwear Sci. 15, S18–S20. 10.1080/19424280.2023.2199263

[B55] Willems T. M. De Clercq D. Delbaere K. Vanderstraeten G. De Cock A. Witvrouw E. (2006). A prospective study of gait related risk factors for exercise-related lower leg pain. Gait Posture 23, 91–98. 10.1016/j.gaitpost.2004.12.004 16311200

[B56] Williams L. R. Standifird T. W. Creer A. Fong H. B. Powell D. W. (2020). Ground reaction force profiles during inclined running at iso-efficiency speeds. J. Biomech. 113, 110107. 10.1016/j.jbiomech.2020.110107 33181396

[B57] Xie P.-P. István B. Liang M. (2022). Sex-specific differences in biomechanics among runners: a systematic review with meta-analysis. Front. Physiol. 13, 994076. 10.3389/fphys.2022.994076 36213228 PMC9539551

[B58] Yang Z. Cui C. Zhou Z. Zheng Z. Yan S. Liu H. (2024). Effect of midsole hardness and surface type cushioning on landing impact in heel-strike runners. J. Biomech. 165, 111996. 10.1016/j.jbiomech.2024.111996 38377740

[B59] Ye D. Li L. Zhang S. Xiao S. Sun X. Wang S. (2024). Acute effect of foot strike patterns on *in vivo* tibiotalar and subtalar joint kinematics during barefoot running. J. Sport Health Sci. 13, 108–117. 10.1016/j.jshs.2023.05.002 37220811 PMC10818114

[B60] Zandbergen M. A. Buurke J. H. Veltink P. H. Reenalda J. (2023). Quantifying and correcting for speed and stride frequency effects on running mechanics in fatiguing outdoor running. Front. Sports Act. Living 5, 1085513. 10.3389/fspor.2023.1085513 37139307 PMC10150107

